# The Role of the Right Hemisphere White Matter Tracts in Chronic Aphasic Patients After Damage of the Language Tracts in the Left Hemisphere

**DOI:** 10.3389/fnhum.2021.635750

**Published:** 2021-06-22

**Authors:** Evie Kourtidou, Dimitrios Kasselimis, Georgia Angelopoulou, Efstratios Karavasilis, Georgios Velonakis, Nikolaos Kelekis, Ioannis Zalonis, Ioannis Evdokimidis, Constantin Potagas, Michael Petrides

**Affiliations:** ^1^Neuropsychology and Language Disorders Unit, Eginition Hospital, First Department of Neurology, National and Kapodistrian University of Athens, Athens, Greece; ^2^Second Department of Radiology, National and Kapodistrian University of Athens, General University Hospital “Attikon”, Haidari, Greece; ^3^Eginition Hospital, Neuropsychological Laboratory, First Department of Neurology, National and Kapodistrian University of Athens, Athens, Greece; ^4^Montreal Neurological Institute, McGill University, Montreal, QC, Canada

**Keywords:** chronic aphasia, right hemisphere, language performance, diffusion tensor imaging (DTI), temporo-frontal extreme capsule fasciculus (TFexcF), arcuate fasciculus (AF), superior longitudinal fasciculus II (SLF II), superior longitudinal fasciculus III (SLF III)

## Abstract

The involvement of the right hemisphere (RH) in language, and especially after aphasia resulting from left hemisphere (LH) lesions, has been recently highlighted. The present study investigates white matter structure in the right hemisphere of 25 chronic post-stroke aphasic patients after LH lesions in comparison with 24 healthy controls, focusing on the four cortico-cortical tracts that link posterior parietal and temporal language-related areas with Broca’s region in the inferior frontal gyrus of the LH: the Superior Longitudinal Fasciculi II and III (SLF II and SLF III), the Arcuate Fasciculus (AF), and the Temporo-Frontal extreme capsule Fasciculus (TFexcF). Additionally, the relationship of these RH white matter tracts to language performance was examined. The patients with post-stroke aphasia in the chronic phase and the healthy control participants underwent diffusion tensor imaging (DTI) examination. The aphasic patients were assessed with standard aphasia tests. The results demonstrated increased axial diffusivity in the RH tracts of the aphasic patients. Patients were then divided according to the extent of the left hemisphere white matter loss. Correlations of language performance with radial diffusivity (RD) in the right hemisphere homologs of the tracts examined were demonstrated for the TFexcF, SLF III, and AF in the subgroup with limited damage to the LH language networks and only with the TFexcF in the subgroup with extensive damage. The results argue in favor of compensatory roles of the right hemisphere tracts in language functions when the LH networks are disrupted.

## Introduction

Research in the 19th century established that language function, such as speech production and comprehension, is critically dependent on specific areas of the left hemisphere (Broca, 1861; see Petrides, 2014). It is now clear that the left hemisphere (LH) is the dominant one for language processing in 95–99% of right-handed individuals and in 70% of left-handed individuals (Rasmussen and Milner, 1975). Brodmann areas 44 and 45 which occupy the cortex of the pars opercularis and pars triangularis of the inferior frontal gyrus, respectively, are often referred to as Broca’s area and play a critical role in the planning and execution of language production in coordination with specific posterior parietal and temporal areas *via* specific bi-directional fasciculi (Indefrey and Levelt, 2004; Petrides, 2015; Sarubbo et al., 2020). In this context, converging evidence from lesion, electrical stimulation, and functional magnetic resonance imaging (fMRI) studies suggests that the posterior superior temporal region provides language-related auditory information *via* the arcuate fasciculus (AF), the supramarginal gyrus of the inferior parietal lobule provides higher level orofacial information *via* the third branch of the superior longitudinal fasciculus (SLF III), and the angular gyrus visuospatial information *via* the second branch of the superior longitudinal fasciculus (SLF II). These pathways are often referred to as the dorsal route or network for language production (Price, 2000; Hickok and Poeppel, 2004; Saur et al., 2008). Access to the meaning of words (semantic processing) has been related to areas in the anterior to the intermediate temporal cortex (Price, 2000; Indefrey and Levelt, 2004; Patterson et al., 2007; Schwartz et al., 2009) and this semantic network interacts with the inferior frontal gyrus, and more specifically area 45, *via* the temporo-frontal extreme capsule fasciculus (TFexcF; see Frey et al., 2008; Saur et al., 2008; Petrides, 2014, 2015). A recent combined DTI and fMRI study by Saur et al. (2008) has provided evidence for the dorsal network in a repetition task measuring sound-to-articulation processes and the ventral temporo-frontal pathway in a listening to meaningful sentences task requiring processing of sound-to-meaning. The latter pathway originates from the intermediate temporal region and continues forward towards the frontal lobe *via* the extreme capsule at the most anterior part of the fronto-temporal bridge known as the temporal stem (Petrides, 2014). It thus links various frontal areas (including Brodmann area 45 which is a major part of Broca’s area in the inferior frontal gyrus) with the lateral temporal cortex involved in sound to meaning relations (Petrides, 2014). Resting–state connectivity studies have also demonstrated this tight coupling between frontal area 45 and the lateral temporal cortex (Margulies and Petrides, 2013). Similarly, reading words, after initial processing within occipitotemporal areas such as in posterior fusiform and lingual gyri, has been related to activation in the left inferior temporal gyrus and left posterior superior temporal gyrus that are considered to comprise the lexical and non-lexical routes for reading (Price, 2000; Devlin et al., 2006; Dien et al., 2013; Sarubbo et al., 2020). The posterior parietal region that has been related to spatial processing and is connected *via* the SLF II with Brodmann area 45 in the inferior frontal gyrus is forming a network that, in the left hemisphere, has been suggested to participate in working memory (Makris et al., 2007), action in space (Koch et al., 2010), and writing (Shinoura et al., 2013).

Note that there is considerable evidence that the right hemisphere (RH) also plays a significant role in language processing. Some of the best evidence comes from studies of epilepsy patients who underwent callosotomy for relief from seizures. Such studies demonstrated that the disconnected RH processes to a variable degree language-related information necessary for word comprehension, spelling, rhyming, object categorization, and exhibits an impressive extent of lexical knowledge (Gazzaniga, 1970, 1998). For example, the RH demonstrated a high level of language comprehension when split-brain patients were instructed *via* dichotic listening to find objects (Milner et al., 1968). Recent studies have also underlined the role of the RH in auditory sentence comprehension *via* the contribution of non-linguistic working memory supported by specific regions such as the right inferior frontal gyrus (Vigneau et al., 2011; Gajardo-Vidal et al., 2018). Split-brain (Levy and Trevarthen, 1977) and lesion (Sidtis et al., 2009) studies have also shown that the RH can produce involuntary, non-propositional speech. Furthermore, certain aspects of language processing, such as the perception and production of prosodic elements (Pell, 1999) and pragmatics (Gernsbacher and Kaschak, 2003), are known to rely largely on the RH function. With the advent of functional neuroimaging, several functional magnetic resonance imaging (fMRI) studies demonstrated that, during the performance of different language tasks, in addition to the expected LH activations, there was often activation in the corresponding RH areas (Price, 2000; Gernsbacher and Kaschak, 2003). Language tasks targeting sentence processing and word retrieval have been shown to evoke bilateral activation in lateral frontal, parietal, and temporal cortical areas (see Bavelier et al., 1997; Gernsbacher and Kaschak, 2003; Vigneau et al., 2011; Price, 2012), demonstrating the contribution of the RH to language function. In addition, there is evidence that after a LH lesion leading to aphasia, tissues in ipsilateral perilesional and contralateral areas are recruited to support recovery (Kiran, 2012; Gainotti, 2015). Saur et al. (2006) showed that after a LH stroke, brain reorganization begins with a strongly reduced activation of remaining left hemisphere language areas in the acute phase, followed by an upregulation with the recruitment of RH homologs of the language zones in the subacute phase, and normalization of activation with a leftward shift in the chronic phase. Indeed, RH recruitment has been found even in the late stages of recovery (Crinion and Price, 2005; Crosson et al., 2009).

There has been considerable discussion regarding the role of this post-stroke right hemisphere activation and its efficacy. Some researchers have argued that RH activation reflects reduced language performance (Postman-Caucheteux et al., 2010; Allendorfer et al., 2012), but others have related RH activation with effective therapeutic intervention (Crosson et al., 2009; Mohr et al., 2014; Kiran et al., 2015). Recently, it has been suggested that a large part of the activation observed in areas of the contralateral right hemisphere does not reflect language-specific activity, but rather an upregulation of activity in intact domain-general systems for cognitive control and attention responding to the increased effort required when domain-specific language networks are impaired (Geranmayeh et al., 2014).

The studies discussed above raise the critical question: Does the brain of chronic aphasic patients exhibit any structural differences related to post-stroke reorganization when compared with the brain of healthy individuals (Kasselimis and Potagas, 2015)? Are these changes restricted to the LH language areas or also include the RH homolog networks? Some studies have reported evidence for gray matter increases to be positively related to better language performance in RH areas of the brain in chronic aphasic patients (Xing et al., 2016; Lukic et al., 2017). Other studies have attempted, with the use of diffusion tensor imaging (DTI), to investigate the structure and role of LH language pathways in aphasic patients, mainly focusing on dorsal fiber tracts, such as the arcuate fasciculus (AF) and the superior longitudinal fasciculus (SLF; Jang, 2013). Those dorsal pathways were related to word/sentence repetition and comprehension, as well as the severity of aphasia and prognosis (Yamada et al., 2007; Breier et al., 2008; Marchina et al., 2011; Kim and Jang, 2013).

More recently, some DTI studies attempted to measure white matter changes in the RH of chronic aphasic patients. Breier et al. (2008) measured the fractional anisotropy (FA) of the right and left superior longitudinal fasciculus (SLF), arcuate fasciculus (AF), and uncinate fasciculus (UF) in 13 patients after left hemisphere cerebrovascular accidents. Although the FA of the left AF and SLF was correlated with speech repetition, no correlation was found between the FA of the homolog fasciculi in the right hemisphere and language performance. There was also no correlation between the right hemisphere FA or the FA of the right AF and language performance of aphasic individuals in the study of Geva et al. (2015) in which the right hemisphere FA did not differ between patients and a control group. However, other studies demonstrated increased volume and number of fibers in the AF (Schlaug et al., 2009) and the UF (Zipse et al., 2012) in aphasic patients after melodic intonation therapy, and these changes were accompanied by improved language performance (Schlaug et al., 2009; Zipse et al., 2012). In the study of Wan et al. (2014), which included non-fluent aphasic patients who had received melodic intonation therapy, reductions were observed in FA of the RH white matter underlying the right inferior frontal gyrus, posterior superior temporal gyrus, and the posterior cingulum. Moreover, speech production improvement was positively associated with a reduction in the FA of the right inferior frontal gyrus (Wan et al., 2014). Interestingly, Pani et al. (2016) observed an increase in the FA underlying the inferior frontal gyrus, and more specifically the pars opercularis, as well as the middle temporal gyrus and precentral gyrus in the right hemisphere of aphasic patients and this increase was positively correlated with speech fluency measures. Forkel et al. (2014), who focused on the arcuate fasciculus, reported that the volume of the long segment of the RH arcuate fasciculus was a predictor of the longitudinal severity in aphasia in patients who suffered a LH stroke.

Most of the above studies focused on DTI metrics, such as tract volume, number of fibers, or FA in dorsal pathways, such as the AF, using a reconstruction protocol by Catani et al. (2005). However, it is now realized that language processing depends not only on the classical dorsal stream which comprises the AF that links the posterior temporal region with Broca’s region and the second and third branches of the superior longitudinal fasciculus (SLF) that link the angular (SLF II) and the supramarginal (SLF III) gyri of the inferior parietal region with Broca’s region (Petrides, 2014). In addition, there is the ventral pathway in the temporal lobe which mediates the interaction between the inferior frontal gyrus (Broca’s area) and anterior to intermediate temporal areas processing semantic information, namely the temporo-frontal extreme capsule fasciculus (TFexcF).

The temporo-frontal extreme capsule fasciculus (TFexcF) was originally discovered in gold standard invasive anatomical tracing studies in the macaque monkey (Petrides and Pandya, 1988, 2009) and was later demonstrated also in the human brain with DTI (Frey et al., 2008; Petrides, 2014). It is a distinct *monosynaptic* bidirectional fasciculus (Petrides and Pandya, 1988, 2009) connecting various frontal areas, including Brodmann area 45 and to a lesser extent Brodmann area 47 with intermediate superior, middle, and inferior temporal areas *via* the extreme capsule (see Petrides, 2014). The TFexcF should not be confused with the inferior fronto-occipital fasciculus (IFOF) which is a multi-component stream of fibers that includes fibers connecting lateral dorsal, medial, and inferior frontal areas with a plethora of regions within temporal and occipital lobes (see Sarubbo et al., 2013; Hau et al., 2016). In this context, some of the monosynaptic direct connectivity between the intermediate temporal cortex and the frontal cortex (i.e., the TFexcF) was in the past included in the reconstructions of the multi-component IFOF. The TFexcF should also not be confused with the UF. The UF connects the temporo-polar cortex and the anterior ventromedial temporal region (e.g., periamygdaloid and entorhinal cortex) with the orbitofrontal cortical region and not the language production areas of the inferior frontal gyrus and has thus been related to emotional regulation and memory processing (Petrides and Pandya, 1988; Ghashghaei and Barbas, 2002; Von Der Heide et al., 2013).

In many tractography studies of aphasic patients, reconstruction protocols that did not distinguish between the different fasciculi of the dorsal stream, namely the AF from the posterior temporal region, the SLF II from the angular gyrus, and the SLF III from the supramarginal gyrus, have been used (Wakana et al., 2004; Catani et al., 2005); and, as pointed out above, in many earlier studies, the ventral stream for language has been attributed to the multi-component stream of fibers that is called the inferior fronto-occipital fasciculus (Martino et al., 2010; Wu et al., 2016; Yang et al., 2017) that comprises fibers linking various occipital areas with temporal areas and even nearby parietal areas which are, in turn, secondarily connected with different frontal areas (Sarubbo et al., 2013; Hau et al., 2016).

The present study investigated in chronic aphasic patients the *structure* of the RH white matter cortico-cortical tracts, which in the LH connect parietal and temporal language-related areas with Broca’s region in the inferior frontal gyrus. These are the dorsal route pathways, namely the AF from the posterior superior temporal region, the SLF II from the angular gyrus, and the SLF III from the supramarginal gyrus of the inferior parietal lobule. Additionally, the present study focused on the TFexcF, namely the ventral pathway that specifically links intermediate temporal cortical regions involved in semantic processing with the inferior frontal gyrus, and is the pathway that corresponds to the findings of fMRI studies that have shown activation of intermediate lateral temporal cortex in relation to the performance of language tasks that require linking sound to meaning (Price, 2000; Indefrey and Levelt, 2004; Saur et al., 2008).

To the best of our knowledge, no previous study had examined with DTI the different fasciculi that include both the dorsal and ventral homologs of the dual route for language in the RH of aphasic patients: the AF, the SLF II, and SLF III in the dorsal route and the TFexcF in the ventral route. Thus, the present study examined in chronic aphasic patients and healthy participants the white matter structure of the RH homologs of the fasciculi that, in the left hemisphere, link Broca’s area in the inferior frontal gyrus with the inferior parietal lobule and the posterior superior temporal region, i.e., the dorsal route (namely AF, SLF II, and SLF III), and the TFexcF that links Broca’s area with the lateral temporal semantic system. In addition, we examined in the chronic aphasic patients the relations of the RH white matter properties of these fasciculi as measured with DTI with performance on language tests. Based on current knowledge of the anatomy of language (see earlier in this section), our hypothesis was that the white matter structure of the right homolog tracts connected with Broca’s area in the inferior frontal gyrus would manifest correlations with language functions to some extent in analogy to what is known regarding the left hemisphere language organization. This hypothesis is based on findings derived from functional MRI studies (Price, 2000, 2012; Crinion and Price, 2005; Crosson et al., 2009; Vigneau et al., 2011; Gajardo-Vidal et al., 2018), split-brain studies (Gazzaniga, 1970, 1998), and DTI studies (Schlaug et al., 2009; Zipse et al., 2012; Pani et al., 2016).

Moreover, one of the aims of the present study was to explore what happens in the right hemisphere of the patients whose left hemisphere lesion was large enough to damage all four tracts examined in this study. For this purpose, the aphasic patients were divided into two subgroups according to whether there was complete (Aphasia Group 1: AG1) or partial (Aphasia Group 2: AG2) damage to the language tracts of interest in the left hemisphere.

An improved methodology in DTI tractography was used that provides distinct reconstruction protocols for the relevant fasciculi and excludes streamlines that belong to neighboring pathways (Badhwar et al., 2016; Barbeau et al., 2020). Furthermore, in addition to FA, DTI scalars that appear to be quite sensitive to a variety of pathological conditions, i.e., DTI metrics such as axial diffusivity (AD) and radial diffusivity (RD) were utilized. These measures can demonstrate specific relationships to white matter pathology (Alexander et al., 2007). Specifically, RD appears sensitive to myelin microstructure and AD to axonal damage (Alexander et al., 2007; Winklewski et al., 2018).

## Materials and Methods

### Participants

Twenty-five (25) right-handed, native, Greek-speaking patients with chronic aphasia after a single left hemisphere middle cerebral artery cerebrovascular accident (CVA) and absence of other neurological or psychiatric conditions were included in the experimental group.

The study included a control group of 24 healthy right-handed adults without any neurological or psychiatric disease, matched with the experimental group (i.e., individuals with aphasia) for age, educational level, and gender. Demographic information, for both the aphasia and control groups, is provided in Table 1. Language function was formally assessed in the individuals with aphasia and all participants underwent MRI examination of the brain. The data of the patients were sampled from the project “Investigation of common anatomical substrates of linguistic and non-linguistic cognitive deficits in post-stroke aphasia” conducted at the Eginition Hospital in Athens, School of Medicine, Greece (research protocol approval ID: ΩΣ3Ξ46Ψ8N2-00Φ, July 2017). Healthy participants were derived from the project “Investigation of cortical surface patterns and their relation with speech metrics and performance in neuropsychological assessment in healthy participants” conducted at the Eginition Hospital in Athens, School of Medicine, Greece (research protocol approval ID: ΩOΞΛ46Ψ8N2-7PN, July 2017). This study was conducted in accordance with the declaration of Helsinki and was approved by the Eginition Hospital Ethics Committee. All participants provided informed consent prior to participation.

**Table 1:** Demographic data for the aphasic and control groups.

	Age Exam	YE	Months Post CVA	M/F
Aphasics *N*=25
Mean (SD)	56.4 (13.2)	13.52 (3.6)	44.8 (39.4)	19 M/6 F
Controls *N*=24
Mean (SD)	53.6 (11.5)	14.4 (2.9)	-	19 M/5 F

*Age Exam, age at examination; CVA, cerebrovascular accident; F, female; M, male; SD, standard deviation; YE, years of formal education*.

### Neuropsychological Evaluation

For the examination of language deficits, the fluency, comprehension, repetition, and reading subtests of the Greek version of the Boston Diagnostic Aphasia Examination—Short Form (BDAE-SF; Tsapkini et al., 2009) were used. Speech fluency was measured by the speech rate (SR) in two tasks (a) the Stroke Story (SS) test, which requires the patient to describe orally her/his stroke incident, and (b) the Cookie Theft Picture (CTP) test, which requires the description of a picture depicting a “cookie theft” event. SR was calculated as the total number of words divided by the total duration of speech (words/minute), as previously implemented in studies investigating narrative abilities in patients with aphasia (see for example: Andreetta et al., 2012; Efthymiopoulou et al., 2017; DeDe and Salis, 2020; Gordon and Clough, 2020; Kasselimis et al., 2020) and patients with primary progressive aphasia (PPA; see for example: Knibb et al., 2009; Wilson et al., 2010). Patients were instructed to speak with no restrictions with respect to narration time. In the case of long silent intervals, the examiners provided the patients with minimal encouragement to continue the narration. In case the patients did not respond, narration assessment was stopped (for a detailed description, see Angelopoulou et al., 2020; Kasselimis et al., 2020).

Additionally, the Greek version of the Boston Naming Test (BNT; Simos et al., 2010) and a receptive vocabulary test, the Greek version of the Peabody Picture Vocabulary Test-Revised (PPVT-R; Simos et al., 2010) were used, including the Controlled Oral Word Fluency (COWF) test (Kosmidis et al., 2004), a verbal fluency task standardized in Greek. The COWF consists of two subscales, one for semantic fluency and one for phonemic fluency. In the semantic fluency subscale, the patient is asked to generate words belonging to a specific category. The three categories are animals, fruits, and objects, and the patient is allowed to generate words for one minute for each category. In the phonemic fluency subscale, the patient is asked to generate words starting with a specific letter of the Greek alphabet. The three letters are “χ” (chi), “Σ” (sigma), and “α” (alpha), and the patient is allowed to generate words for one minute for each letter. Finally, the Comprehension of Instructions test in Greek (CIG; Simos et al., 2014), a verbal comprehension task standardized for the Greek population, was also included. In this test, the stimulus is a plate depicting five crosses and four circles varying in color and arranged in a 3 × 3 grid. The patient is asked to point to two or more shapes in a particular sequence specified by the examiner. Overall, the test includes 14 verbal commands of increasing complexity. Because this test does not require a verbal response, it is considered suitable for testing patients with non-fluent aphasia and therefore restricted speech output. Language performance was not examined in the control group since a ceiling effect has been reported in the performance of healthy individuals on the BDAE Short Form (Tsapkini et al., 2009).

### Magnetic Resonance Imaging Scanning Protocol

All participants underwent the same imaging protocol on a 3T Achieva TX Philips manufactured MRI scanner (Philips, Best, The Netherlands). The acquisition protocol included a sagittal 3D high-resolution T1 (3D-HR-T1) weighted sequence [repetition time (TR): 9.9 ms, time echo (TE): 3.7 ms, flip angle: 70°, acquisition voxel size 1 × 1 × 1 mm], and an axial single-shot spin-echo echo-planar imaging (EPI) sequence with 30 diffusion encoding directions (TR: 7,299 ms, TE: 68 ms, flip angle: 90°, acquisition voxel size: 2 × 2 × 2 mm, sensitivity encoding reduction factor of 2, two b factors with 0 s/mm^2^ (low b) and 1,000 s/mm^2^ (high b).

### Diffusion Tensor Imaging (DTI) Analysis

The DTI image processing and the tract reconstructions were performed with Brainance MD (Advantis Medical Imaging, Eindhoven, The Netherlands). Motion and eddy-current correction was performed with the registration tool of the scanner (Netsch and van Muiswinkel, 2004; Wang et al., 2011). DTI sets underwent a co-registration protocol available in the Brainance MD. For the reconstruction of the selected right and left hemisphere white matter tracts, regions of interest (ROIs) were drawn using the protocols described below. The quantitative measures of the DTI variables calculated were mean fractional anisotropy (FA), axial diffusivity (AD), and radial diffusivity (RD). Angle and FA thresholds were set at 35° and 0.20, respectively.

### Region of Interest (ROI) Protocols

**Figure 1:**
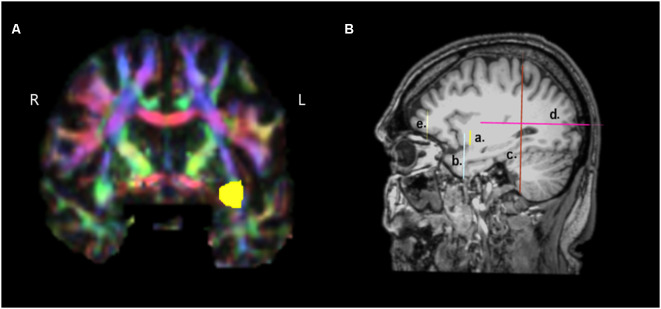
Presentation of the regions of interest (ROIs) used for the reconstruction of the temporo-frontal extreme capsule fasciculus (TFexcF). **(A)** Coronal section at the most anterior point where the temporal lobe joins with the frontal lobe. The green region of the relevant white matter in the right (R) hemisphere is marked with yellow color in the left (L) hemisphere. **(B)** a. Location of the inclusion ROI in the anteriormost joining of temporal lobe with the frontal lobe (see **A**); b. location of the exclusion ROI for the temporal pole and anterior ventromedial temporal lobe; c. location of the exclusion ROI for the occipital region; d. location of the exclusion ROI for the posterior temporoparietal region; e. location of the exclusion ROI for the most anterior region of the frontal lobe.

**Figure 2:**
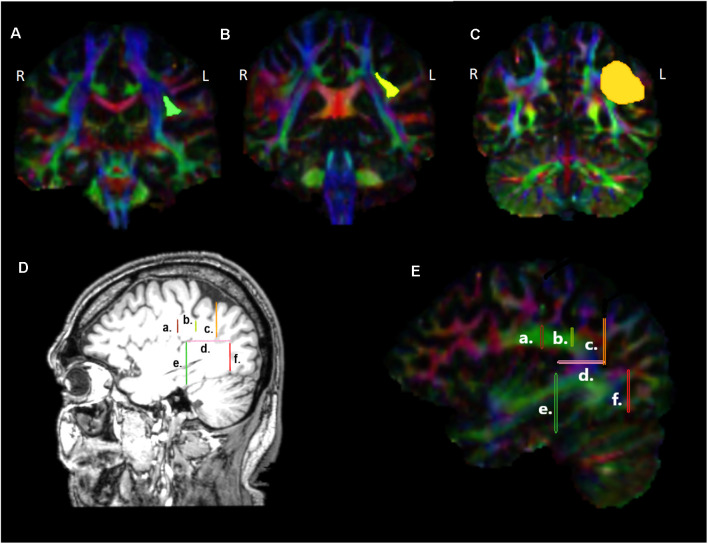
Presentation of the regions of interest (ROIs) used for the reconstruction of the dorsal tracts, namely the arcuate fasciculus (AF), the third (SLF III), and second (SLF II) branches of the superior longitudinal fasciculi in coronal sections **(A–C)**, at locations indicated in **(D)**, **(E)** sagittal sections. a. Location of the first ROI as indicated by the green highlighted region in **(A)**, b. location of the ROI at the level of the supramarginal gyrus, indicated with yellow in **(B)**, c. location of the ROI placed immediately before the angular area, indicated with orange in **(C)**, d. location of the ROI at the temporo-parietal junction, just below the descending posterior ramus of the lateral fissure, e. location of the ROI used for the exclusion of fibers heading towards anterior temporal areas, f. location of the ROI used for the exclusion of fibers heading towards occipital areas. L, left hemisphere; R, right hemisphere.

For the reconstruction of the TFexcF, the protocol described by Frey et al. (2008) and Badhwar et al. (2016) was implemented. The SLF II, the SLF III, and the AF were reconstructed according to the protocols of Barbeau et al. (2020). A detailed presentation of the inclusion and exclusion ROIs used for the reconstruction of the ventral TFexcF and the dorsal pathways, i.e., the AF, SLF III, and SLF II, is displayed in Figures 1, 2, respectively.

For the TFexcF reconstruction, a coronal section at the point where the anterior temporal lobe starts connecting with the frontal lobe (Y+3 in MNI coordinates) is identified and then, in the color map, an inclusion ROI is drawn around the green spot just below the infero-lateral frontal cortex (Figures 1A and Figures 1Ba). After the reconstruction of the fibers passing through that ROI, on a coronal section just anterior to the initial TFexcF ROI, an exclusion ROI that encompasses the entire temporal polar region is created, in order to exclude the fibers of the uncinate fasciculus terminating in the temporal polar region (Figure 1Bb). Subsequently, fibers that pass from the temporal to the occipital and posterior parietal areas are also excluded with an exclusion ROI that includes the entire hemisphere placed on a coronal section at the end of the lateral fissure (Figure 1Bc). Then, fibers passing dorsally to the parietal areas are excluded with the use of an exclusion ROI just above the lateral fissure (Figure 1Bd). Finally, the branch of the tract that proceeds towards orbito-frontal areas, anterior to the pars orbitalis (Figure 1Be), is also excluded.

For the reconstruction of the AF linking the posterior superior temporal region (Wernicke’s area) with Broca’s region, on the color map, the reverse “C” white matter in green is identified to locate the arcuate fasciculus (Figure 2E), and a first ROI is drawn in the coronal section (Figures 2A, 2Da and 2Ea) immediately under the central sulcus, around the green triangle [see Figure 2A highlighted green triangle in the left (L) hemisphere], at around Y-15 in MNI coordinates. A second inclusion ROI was drawn in the axial view at the temporo-parietal junction, just below the descending posterior ramus of the lateral fissure, around the blue area where the vertical part of the arcuate fasciculus lies (Figures 2Dd and 2Ed). An exclusion ROI was added in the coronal view, after the most anterior section where Heschl’s gyrus is visible (Y-31) in order to exclude fibers passing to anterior temporal areas (Figures 2De and 2Ee). A second exclusion ROI was also placed at the border between the temporal gyri and the occipital lobe to exclude fibers ending in the occipital lobe (Figures 2Df and 2Ef) at Y-79.

For the reconstruction of the SLF III linking the supramarginal gyrus with inferior frontal gyrus, in the color map, the same first ROI used for the reconstruction of the AF was initially selected (Figures 2A, 2Da and 2Ea). Then, an inclusion ROI was placed around the green triangle, at the level of the supramarginal gyrus, at around Y-39 (see the yellow area in 2B, 2Db and 2Eb). For the exclusion of the fibers heading posteriorly towards the angular gyrus an exclusion ROI was drawn at the point where the angular gyrus begins (Y-55; Figures 2C, 2Dc and 2Ec). Finally, fibers passing to the temporal areas were excluded with the use of an exclusion ROI placed on an axial view, below the supramarginal and angular gyri (Figures 2Dd and 2Ed).

For the reconstruction of the SLF II that links the angular gyrus with the inferior frontal region, the ROI located at the reverse “C” below the central sulcus (Figures 2A, 2Da, 2Ea) as well as the ROI located just before angular gyrus (Figures 2C, Figures 2Dc, 2Ec) were used as inclusion ROIs, while fibers heading towards the temporal lobe were excluded with the use of the ROI located at the level of the temporoparietal gyrus (Figures 2Dd and 2Ed).

**Figure 3:**
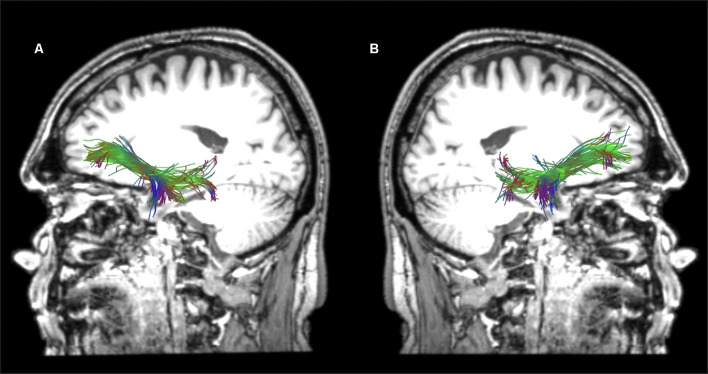
Sagittal views of the reconstructed temporo-frontal extreme capsule fasciculus (TFexcF) in the left **(A)** and right **(B)** hemispheres of a healthy participant in native space.

**Figure 4:**
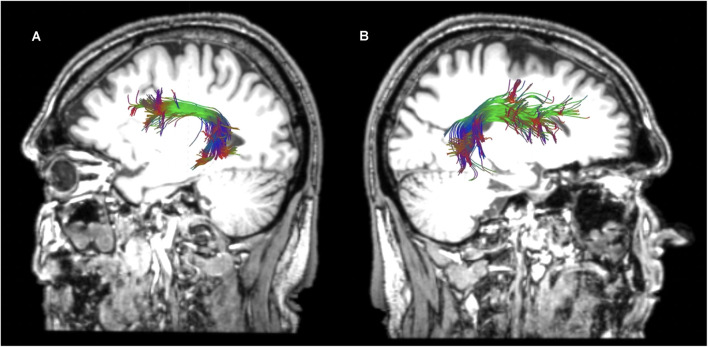
Sagittal views of the reconstructed arcuate fasciculus (AF) in the left **(A)** and right **(B)** hemispheres of a healthy participant in native space.

**Figure 5:**
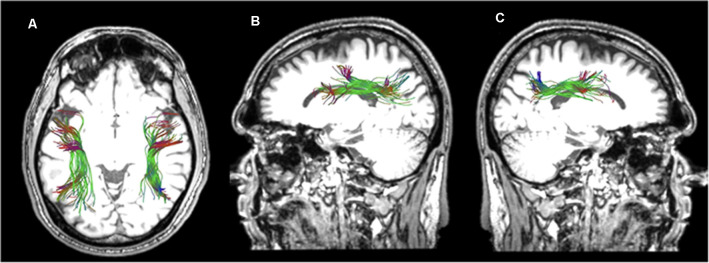
Horizontal **(A)** and sagittal views of the reconstructed left **(B)** and right **(C)** superior longitudinal fasciculus II of a healthy participant in native space.

**Figure 6:**
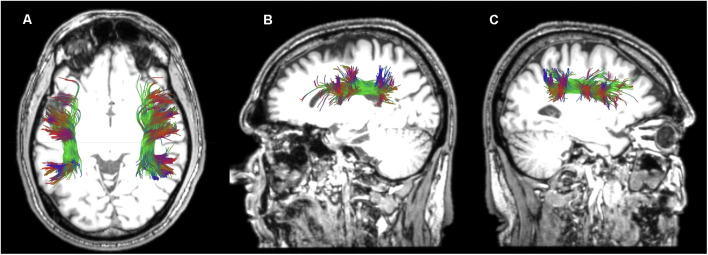
Horizontal **(A)** and sagittal views of the reconstructed left **(B)** and right **(C)** superior longitudinal fasciculus III of a healthy participant in native space.

Reconstructions of the selected tracts in a healthy participant are presented in Figures 3–6. In the cases where the lesion was present, and thus the anatomical landmarks or the color map could not be used to define the precise location of the ROIs, the ROIs were drawn by analogy to the intact hemisphere. Additionally, ROIs were placed in adjacent sections to ensure that reconstructions were as precise as possible.

### Data Processing Analysis

After the reconstruction of the LH white matter tracts, the experimental group was further divided into individuals with aphasia in whom there was an absence of reconstruction of the left hemisphere language pathways because of the extensive lesion [henceforth AG1 (Aphasia Group 1); *n* = 8] and individuals with aphasia in whom there was partial damage to the left hemisphere language pathways [henceforth AG2 (Aphasia Group 2); *n* = 17], as described later in this section. In order to check whether the possible right hemisphere white matter changes were related to the occurrence of aphasia and the process of recovery, an additional right hemisphere white matter tract, which is not considered as a typical language-related tract, namely, the right uncinate fasciculus (UF) was reconstructed, measured, and compared between the aphasia group and the control group. Note that the UF connects orbitofrontal and ventromedial frontal areas with the temporal pole and anterior ventromedial temporal lobe (e.g., amygdala, pyriform, entorhinal cortex, etc.) and is involved in the regulation of emotional and memory processes (Von Der Heide et al., 2013).

Since FA, AD, and RD values were normally distributed and equal variance was confirmed, independent samples *t*-test was conducted to compare the differences of these DTI variables of the right hemisphere white matter tracts between all the individuals with aphasia (*n* = 25) and the controls (*n* = 24), with the use of SPSS (V.25). Because the group of patients was further separated into two smaller groups (the AG1 and AG2 subgroups), we conducted Kruskal-Wallis tests to compare FA, RD, and AD values of the RH white matter tracts between the two aforementioned aphasic sub-groups and the controls. After pairwise comparisons using Mann-Whitney tests, corrections for multiple comparisons were conducted using false discovery rate (FDR; Benjamini and Hochberg, 1995). Furthermore, we investigated the relationship between language performance and the structure of the RH white matter tracts in AG1, namely the group with complete damage of the language-related tracts in the left hemisphere. Since language performance data did not follow the normal distribution and *n* < 30, the Spearman Rho correlation coefficient was used. The Spearman Rho between the structure of the RH white matter tracts and language performance was also conducted for the AG2 subgroup. Again, *p-values* were adjusted with FDR. The strength of the observed correlations was characterized according to Ratner (2009). Additionally, the Mann–Whitney test was conducted in order to compare the language performance between the two aphasia subgroups (i.e., AG 1 and AG2).

### Intra-rater and Inter-rater Reliability

All DTI data were analyzed twice, with the second analysis taking place one month after the first analysis, by a single rater (EKo) who was unaware of the language performance of each participant. The placement of ROIs and the reconstructed tracts were supervised by an experienced neuroanatomist (MP). The datasets of 10 participants were also analyzed by a second rater (GA) who was unaware of the results of the first rater.

### Reconstruction of the Left Hemisphere Language Tracts

The extensive total lesion volume in the left hemisphere made impossible the reconstruction of the AF, SLF II, SLF III, and TFexcF in eight patients in the aphasia group. These patients composed the AG1 aphasia subgroup. In the remaining 17 patients, who composed the AG2 aphasia subgroup, one, two, or three of the selected left language-related pathways could not be reconstructed, and in the patients in whom all four fasciculi were reconstructed, at least one of these manifested significantly reduced FA (Table 2), compared to healthy participants (Table 3). Mann–Whitney tests between the two aphasia subgroups (i.e., AG1 and AG2) showed that the total lesion volume differed significantly (*p* < 0.001), with the AG1 group presenting increased lesion volume (mean 210.08 cc and standard deviation 60.70) compared to AG2 (mean 45.00 cc and standard deviation 29.54).

**Table 2:** Lesion volume in cubic centimeters and FA values calculated for the left hemisphere TFexcF, SLF III, AF, and SLF II of the patients with aphasia.

Patient	L TFexcF	L SLF III	L AF	L SLF II	Lesion volume (cc)
A2	-	-	-	-	238.26
A5	-	-	-	-	181.66
A6	-	-	-	-	182.87
A10	-	-	-	-	278.57
A11	-	-	-	-	260.82
A21	-	-	-	-	221.28
A24	-	-	-	-	231.92
A22	-	-	-	-	85.26
A1	0.3434*	-	-	-	65.69
A12	0.4324	0.3549*	-	-	69.95
A4	0.3553	0.3571*	-	-	12.62
A25	0.4218	0.3745*	-	-	29.61
A7	0.4122	0.3842*	0.4129*	-	74.69
A16	0.3659*	0.3882*	0.403*	-	51.52
A19	0.4483	0.3650*	0.3391*	-	47.74
A15	0.2813*	0.3428*	-	0.3421*	52.55
A8	-	0.4151	0.4142*	0.4276	7.84
A9	-	0.4285	0.4985	0.4399	35.53
A13	-	0.4227	0.443*	0.4187	46.38
A14	-	0.4301	0.4542	0.4145	21.80
A17	0.4147*	0.3578*	0.3765*	0.3957*	5.97
A18	0.4563	0.3397*	0.3570*	0.3951*	18.67
A20	0.4771	0.4247	0.4104*	0.4229	26.66
A3	0.42	0.3568*	0.3628*	0.3528*	58.65

*Fractional anisotropy (FA) values 3 SD below the mean FA of the control group, as presented in Table 3. (-) Reconstruction failure of the tracts affected by the left hemisphere lesion. AF: Arcuate Fasciculus; SLF II: Superior Longitudinal Fasciculus II; SLF III: Superior Longitudinal Fasciculus III; TFexcF: Temporo-Frontal extreme capsule Fasciculus.

**Table 3:** Mean FA and standard deviation (SD) of the left (L) hemisphere language tracts in the healthy participants (i.e., the control group).

	Mean FA	SD
L TFexcF FA	0.4486	0.0165
L SLFIII FA	0.4538	0.0177
L SLFII FA	0.4372	0.0204
L AF FA	0.4871	0.0165

*AF: Arcuate Fasciculus; FA: fractional anisotropy; SLF II: Superior Longitudinal Fasciculus II; SLF III: Superior Longitudinal Fasciculus III; TFexcF: Temporo-Frontal extreme capsule Fasciculus*.

### Left Hemisphere Lesion Description

**Figure 7:**
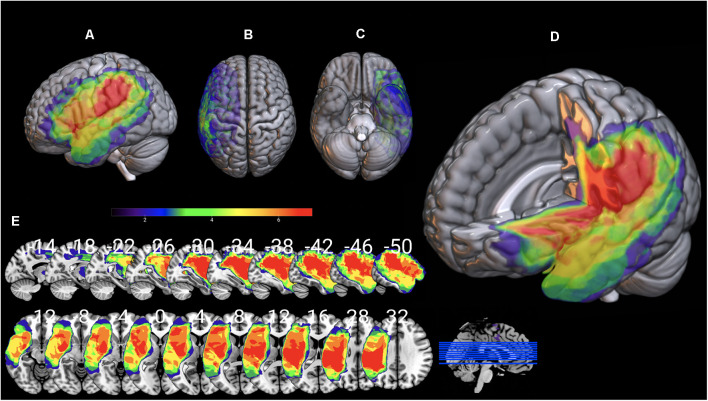
Lesion maps overlay for the AG1 group are shown on 3D views of the standard average brain. The left hemisphere **(A)** and the dorsal **(B)** and orbital **(C)** views of the brain are presented. In **(D)** part of the left hemisphere is removed. The sagittal and axial serial sections displaying the lesions in MNI stereotaxic coordinates are shown in **(E)**.

**Figure 8:**
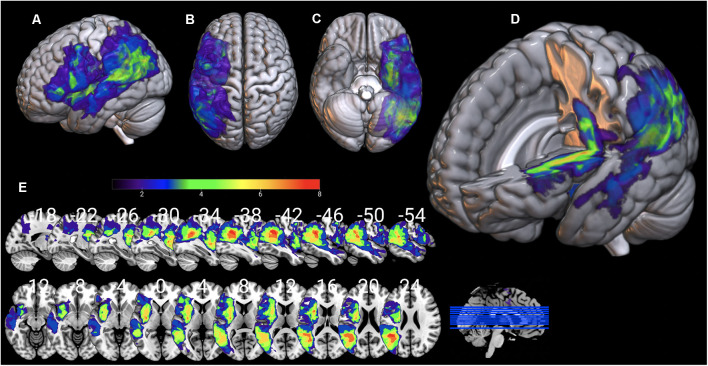
Lesion maps overlay for the AG2 group are shown on 3D views of the standard average brain. The left hemisphere **(A)** and the dorsal **(B)** and orbital **(C)** views of the brain are presented. In **(D)** part of the left hemisphere is removed. The sagittal and axial serial sections displaying the lesions in MNI stereotaxic coordinates are shown in **(E)**.

Individual 3D T1 images were used to draw manually brain lesions in MRIcron (Rorden and Brett, 2000) by two experienced neuropsychologists (DK and GA), prior to the cognitive assessment of the patients. All lesion maps have been visually inspected by an experienced neurologist (CP) blind to the patients’ language profile. T1 and lesion maps were then normalized on SPM12, using Clinical toolbox, a tool designed for brain injury imaging data pre-processing, using SPM12’s unified segmentation–normalization and implementing age specialized templates (younger and older adults; see Rorden et al., 2012 for a detailed description) and total lesion volume was extracted. Lesion overlap for the AG1 and AG2 sub-groups is presented in Figures 7, 8 in MRIcronGL^1^.

## Results

### Right Hemisphere White Matter Tract Comparison and Correlations With Language Performance

*T*-test analysis between the control subjects (*n* = 24) and individuals with aphasia (*n* = 25) showed that the two groups differed significantly in all dorsal fasciculi examined and only marginally in the ventral TFexcF. In particular, after applying FDR corrections for multiple comparisons, the two groups differed significantly in: (a) R AF AD (*p*_adjusted_ = 0.009), (b) R SLF III AD (*p*_adjusted_ = 0.014), (c) R SLF II AD (*p*_adjusted_ = 0.014) and R SLF II RD (*p*_adjusted_ = 0.016), and marginally in R TFexcF AD (*p*_adjusted_ = 0.068). In all tracts, AD values, as well as the RD of the R SLF II, were higher for the patient group compared with the controls. By contrast, none of the tracts examined showed other significant RD (R TFexcF RD *p*_adjusted_ = 0.182; R SLF III RD *p*_adjusted_ = 0.166; R AF RD *p*_adjusted_ = 0.078) or FA differences (R TFexcF FA *p*_adjusted_ = 0.905; R SLF III FA *p*_adjusted_ = 0.484; R SLF II FA p_adjusted_ = 0.359; R AF FA *p*_adjusted_ = 0.610). Regarding the DTI values of the right hemisphere uncinate fasciculus (R UF), no significant or marginally significant differences were observed between the two groups (R UF FA *p*_adjusted_ = 0.484; R UF AD *p*_adjusted_ = 0.093; R UF RD *p*_adjusted_ = 0.101).

Additionally, comparisons between the control group and the two sub-groups of patients, using Kruskal–Wallis and Mann–Whitney *post-hoc* analysis, did not reveal any statistically significant differences between the two aphasia groups, after applying FDR corrections for multiple comparisons. The aforementioned non-parametric analysis generally confirmed the results of the *t*-tests, indicating differences between the patient group and healthy individuals. In summary, patients with aphasia differed from the control group, but the two aphasia subgroups were comparable, with regard to several anatomical indices (see Table 4). Mean FA, AD, and RD values for the control group, the aphasia group, as well as the two patient subgroups (i.e., AG1 and AG2) are presented in Table 5.

**Table 4:** Individual scores of patients’ neuropsychological performance.

	AC-W	AC-SC	AC-CM	Rp-W	Rp-S	Rd-W	Rd-S	Rd-SCa	Rd-SCb	COWF-ph	COWF-s	BNT	PPVT-R	CIG	Aphasia severity
**AG1**													
P1	161	9*	61	4	4*	151*	3*	1*	4	12*1	28*1	171	291	3*	Moderate
P2	11*	3*	1*	0*	0*	0*	0*	0*	0*	0*	0*	5*	22*	1*	Severe
P3	7*	0*	1*	0*	0*	0*	0*	0*	0*	0*	0*	0*	2*	0*	Severe
P4	141	9*	2*	3*	0*	3*	0*	3*	3	5*	9*	91	NA	NA	Severe
P5	161	7*	61	5	5*	7*	0*	3*	4	3*	8*	121*	NA	3*	Severe
P6	11*	1*	1*	1*	2*	0*	0*	0*	0*	0*	0*	2*	101	0*	Severe
P7	4*	1*	0*	0*	0*	0*	0*	0*	1*	0*	0*	0*	9*	0*	Severe
P8	161	5*	3*	4	101*	151*	3*	31	4	0*	21*1	6*	15*	1*	Moderate
**AG2**													
P9	141	8*	4*	5	101*	141	1*	2*	4	6*	17*1	151	19*	0*	Moderate
P10	151	2*	0*	2*	2*	151	5	2*	4	1*	0*	14*	18*	NA	Moderate
P11	161	8*	51	5	101	151	4	2*	4	18*1	321*	181	23	3*	Mild
P12	151	101	61	4	101	151	5	2*	4	261*	531*	201	31	9*	Mild
P13	14.5*	91	61	5	9	12*	0*	2*	4	0*	0*	6*	28	3*	Moderate
P141	15.5	91	3*	5	101	151	3*	1*	4	10*1	311*	181	30	5*	Moderate
P151	12.5	81	61	5	101	NA	NA	NA	2*	18*1	3*	8*	24	6*	Moderate
P16	10.5*	5*	1*	3*	0*	151	2	31	4	0*	221*	9*	17	0*	Moderate
P17	151	6*	51	4	101	12*	1*	2*	2*	NA	NA	9*	NA	NA	Moderate
P18	151	101	51	3*	9	5*	0*	1*	3	2*	19*1	6*	NA	NA	Moderate
P19	5*	0*	0*	2*	0*	0*	0*	0*	0*	0*	0*	0*	2*	0*	Moderate
P20	161	8*	61	4	2*	151	5	31	4	201*	331*	161	24	4*	Mild
P21	161	101	61	5	101	151	5	31	4	291*	541*	201	28	131	Mild
P22	141	7*	4*	4	3*	9*	1*	2*	NA	0*	9*	3*	12	1*	Moderate
P23	151	101	51	5	101	151	4	2*	4	12*1	391*	191	25	7	Moderate
P24	151	101	51	5	101	151	4	31	4	11*1	321*	171	20	6*	Mild
P25	161	101	61	5	9	151	5	1*	4	14*1	8*	4*	23	3*	Moderate

AC–W: BDAE Auditory comprehension—single words; AC–SC: BDAE auditory comprehension—simple commands; AC–CM: BDAE Auditory comprehension—complex material; Rp–W: BDAE word repetition; Rp–S: BDAE sentence repetition; Rd–W: BDAE single word reading; Rd–S: BDAE Sentence reading; Rd–SC: BDAE sentence comprehension; COWF–ph: Controlled Oral Word Fluency phonemic subscale; COWF–s: Controlled Oral Word Fluency semantic subscale; BNT: Boston Naming Test; PPVT-R: Peabody Vocabulary Test-Revised; CIG: Comprehension of Instructions in Greek. *Impaired scores based on the corresponding adaptation/normative studies and/or clinical judgement.

**Table 5:** Mean and standard deviations for FA, AD, and RD values of the right hemisphere UF, TFexcF, AF, SLF III, and SLF II white matter tracts for all participants.

	Patients (*n* = 25)		Controls (*n* = 24)		AG1 patients (*n* = 8)		AG2 patients (*n* = 17)	
	Mean	SD	Mean	SD	Mean	SD	Mean	SD
UF FA	0.40839	0.02087	0.41444	0.01855	0.39897	0.01833	0.41282	0.021
UF AD	0.0012	0.00004	0.00118	0.00003	0.001177	0.00004	0.00121	0.00004
UF RD	0.000617	0.00003	0.000599	0.00002	0.0006174	0.00003	0.0006173	0.00003
TFexcF FA	0.42398	0.02934	0.42583	0.02111	0.4097	0.02304	0.43198	0.02983
TFexcF AD	0.001202	0.00005	0.001176	0.00003	0.001186	0.00001	0.00121	0.00006
TFexcF RD	0.000607	0.00004	0.000591	0.00002	0.000614	0.00002	0.000604	0.00002
AF FA	0.43902	0.03239	0.44492	0.02002	0.45125	0.02872	0.43327	0.0332
AF AD	0.001169	0.00005	0.001129	0.00003	0.001171	0.00003	0.001169	0.00005
AF RD	0.000575	0.00004	0.000549	0.00002	0.000557	0.00003	0.000583	0.00005
SLF III FA	0.430102	0.02292	0.43576	0.02363	0.43743	0.01667	0.42665	0.02504
SLF III AD	0.0011523	0.00004	0.0011157	0.00003	0.0011493	0.00003	0.0011537	0.00005
SLF III RD	0.000605	0.00001	0.000558	0.00002	0.000643	0.0002185	0.0005874	0.00004
SLF II FA	0.42816	0.02055	0.43589	0.02142	0.43472	0.01803	0.42508	0.02145
SLFII AD	0.001155	0.00004	0.001119	0.00003	0.001153	0.00003	0.001156	0.00005
SLF II RD	0.000581	0.00003	0.000554	0.00002	0.00057	0.00002	0.000586	0.00003

*AF: Arcuate Fasciculus; AD: axial diffusivity; FA: fractional anisotropy; RD: radial diffusivity; SLF II: Superior Longitudinal Fasciculus II; SLF III: Superior Longitudinal Fasciculus III; TFexcF: Temporo-Frontal extreme capsule Fasciculus; UF: Uncinate Fasciculus*.

**Figure 9:**
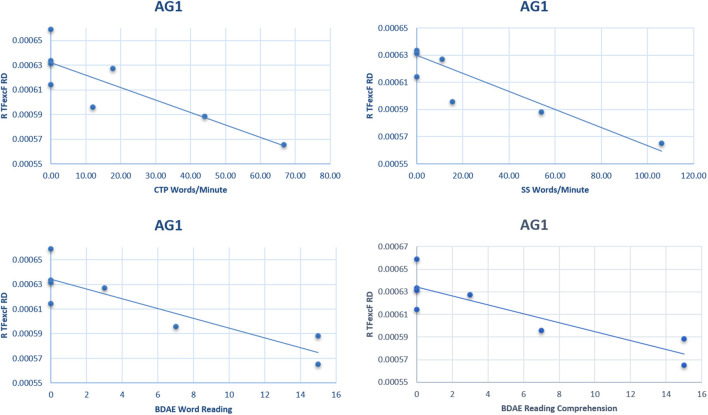
Scatterplots of the significant correlations between language performance and radial diffusivity (RD) of the right hemisphere TFexcF in the AG1 subgroup. BDAE, Boston Diagnostic Aphasia Examination; CTP, Cookie Theft Picture test; SS, Stroke Story test.

The correlation analysis between the language performance of AG1 (in which none of the LH language tracts could be reconstructed because of the presence of the extensive brain lesion) with the RH white matter DTI values, after the FDR corrections, indicated a strong negative relationship between the right hemisphere TFexcF RD and (a) reading words (BDAE; ρ = −0.899, *p*_adjusted_ = 0.016); (b) reading comprehension (BDAE; ρ = −0.877, *p*_adjusted_ = 0.045); (c) number of words/minute during the Stroke Story Test (BDAE; ρ = −0.824, *p*_adjusted_ = 0.054); and (d) number of words/minute during the Cookie Theft Picture Test (BDAE; ρ = −0.824, *p*_adjusted_ = 0.054). Scatterplots of the significant correlations observed in the AG1 subgroup between language performance and RD are presented in Figure 9.

On the other hand, in the AG2 subgroup (in which the left language network was partly damaged), the correlation between language performance and RH white matter tract structure was observed both in the ventral TFexcF as well as the dorsal AF and SLF III RH tracts. Specifically,

(1) A significant moderate negative correlation was observed between the right hemisphere TFexcF RD and (a) simple command comprehension (BDAE; ρ = −0.641, *p*_adjusted_ = 0.026); (b) word repetition (BDAE; ρ = −0.699, *p*_adjusted_ = 0.012); (c) PPVT-R (ρ = −0.693, *p*_adjusted_ = 0.018), as well as a fairly strong negative correlation with (d) sentence repetition (BDAE; ρ = −0.714, *p*_adjusted_ = 0.012); and (e) CIG (ρ = −0.748, *p*_adjusted_ = 0.012).
(2) The right hemisphere SLF III RD showed a significant moderate negative correlation with word repetition (BDAE; ρ = −0.632, *p*_adjusted_ = 0.042), and PPVT-R (ρ = −0.662, *p*_adjusted_ = 0.026), as well as a strong negative correlation with sentence repetition (ρ = −0.768, *p*_adjusted_ < 0.001). A marginally significant negative correlation was also observed with (a) phonemic fluency (ρ = −0.593, *p*_adjusted_ = 0.051); (b) BNT (ρ = −0.555, *p*_adjusted_ = 0.051); and (c) CIG (ρ = −0.604, *p*_adjusted_ = 0.051).
(3) The right SLF III FA was strongly and positively related to sentence repetition (BDAE; *ρ* = 0.714, *p*_adjusted_ = 0.018), and
(4) The right AF RD manifested a strong negative correlation with sentence repetition (BDAE; ρ = −0.704, *p*_adjusted_ = 0.036) and moderate negative correlation with word repetition (BDAE; ρ = −0.664, *p*_adjusted_ = 0.036).

**Figure 10:**
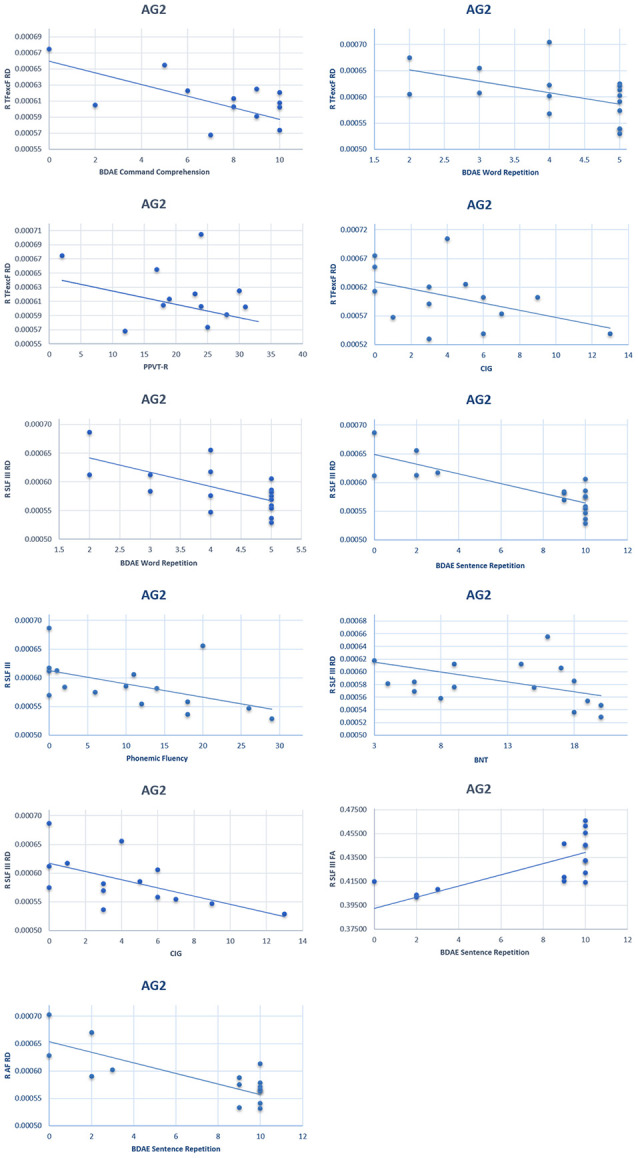
Scatterplots of the significant correlationsbetween language performance and radial diffusivity/fractional anisotropy of the right hemisphere TFexcF, SLF III, and AF in the AG2 subgroup. BDAE: Boston Diagnostic Aphasia Examination; PPVT-R: Peabody Vocabulary Test-Revised; CIG: Comprehension of Instructions Test in Greek; BNT: Boston Naming Test.

Correlation coefficients indicating associations between anatomical white matter indices and cognitive scores are presented in Supplementary Table 1. Scatterplots of the significant correlations observed in the AG2 subgroup between language performance and DTI values described above are presented in Figure 10.

Moreover, Mann–Whitney *U* tests were conducted in order to compare performance in language testing between the two subgroups of patients. *P*-values were corrected using FDR. Results indicated significant differences in the repetition of words (*p*_adjusted_ = 0.05) and sentences (*p*_adjusted_ = 0.05), in reading sentences (*p*_adjusted_ = 0.05) and in speech rate in stroke story narration (*p*_adjusted_ = 0.008) and cookie theft picture description (*p*_adjusted_ < 0.001), with AG2 being superior.

## Discussion

After a left hemisphere CVA lesion and resulting aphasia, intact areas in the LH and/or the RH are recruited to compensate for the loss of language function (Hartwigsen and Saur, 2019). Although the RH is considered to be the "minor hemisphere" for language, it does contribute significantly to language processing, and, in many patients with severe aphasia, it is the brain region for any remaining language processing. The language production region known as Broca’s area (Brodmann areas 44 and 45) in the inferior frontal gyrus is bidirectionally linked with the posterior superior temporal region (Wernicke’s region) *via* the AF and the adjacent supramarginal and angular gyri of the inferior parietal lobule *via* SLF III and SLF II, respectively. The functional interaction of Broca’s area with the anterior to intermediate temporal cortical region that processes semantic information takes place *via* the TFexcF. The present study investigated the structural properties of the homologs of these four language-related fasciculi in the RH of chronic aphasic patients.

The findings indicate that the chronic aphasic patients, in comparison to the control subjects, demonstrate significant structural differences in the right homologs of the language-relevant tracts examined: higher AD in all RH dorsal tracts (i.e., the AF, SLF II, and SLF III) and marginally higher AD in the ventral TFexcF, as well as higher RD in the right hemisphere SLF II. RD is a measure of water diffusion perpendicular to the axon and has been quite consistently related to myelin microstructure (Alexander et al., 2007). Specifically, higher RD has been related to increased axonal demyelination (Alexander et al., 2007; Winklewski et al., 2018). AD, on the other hand, is a measure of water diffusion parallel to the axonal course and has been related to axonal status (Alexander et al., 2007). Decreased AD has been related to acute axonal damage (Song et al., 2003) and has been shown to predict aphasia and motor outcome 3 months post-stroke (Moulton et al., 2019). Moreover, AD increases have also been related to axonal damage (Arfanakis et al., 2002), as has been shown in the study of Kraus et al. (2007) who examined the white matter in chronic traumatic brain injury (TBI) patients. However, as Winklewski et al. (2018) noted in their review “pathologic changes from acute to chronic stage result in axial diffusivity becoming less informative over time,” leading to limited correlations of AD with performance. Thus, according to the above, and keeping in mind that all of the aforementioned DTI studies focus on the white matter *directly* affected by a certain pathological mechanism, the present study cannot differentiate between the causes of the observed differences in AD manifested in the non-lesioned right hemisphere of the aphasic patients compared to the healthy participants. Based on the existing literature, these differences could also reflect indirect effects of pathological mechanisms, such as edema (Schallert et al., 2000; Johansson, 2004; Winklewski et al., 2018).

Interestingly, as reported in the results section, it is not the AD measures in the RH of the patients that correlated with language performance, but, rather, the RD measures. There were significant negative correlations between increased RD and language performance. Thus, lower myelination properties, as indicated by the increased RD, were associated with lower language performance. Since the specific RD measures shown to correlate with performance in the patients did not differ significantly in comparison with the healthy individuals, our hypothesis is that premorbid RH white matter structure and, in particular, myelination variability could be important for post-stroke language performance.

A few investigations had previously attempted to examine possible language-related white matter structure in the intact hemisphere of aphasic patients, focusing mostly on the FA or tract volume. Some of these studies failed to observe RH differences in the AF in aphasic patients compared to controls (Breier et al., 2008; Geva et al., 2015), while others reported increased AF volume after melodic intonation therapy and improved performance (Schlaug et al., 2009; Zipse et al., 2012). Pani et al. (2016) related speech fluency to higher FA in the white matter below the precentral gyrus, superior temporal gyrus, and Brodmann area 44 of the inferior frontal gyrus in the RH. In another study, a reduction of FA was reported in the right hemisphere below the inferior frontal gyrus and the posterior superior temporal gyrus in non-fluent patients after speech therapy (Wan et al., 2014). However, note that none of these earlier studies had used protocols that permitted exploration of the separate contributions of SLF II, SLF III, and AF, namely the three distinct fasciculi that constitute the dorsal route for language processing and which are related to different aspects of sound to articulation mapping. These three fasciculi connect distinct cortical regions (Petrides, 2014) and require appropriate paradigms to assess their specific roles.

One of the key questions explored in the present investigation was the relation with language performance of the right hemisphere homologs of the four critical left hemisphere language-related fasciculi in patients whose LH lesion had resulted in the complete destruction of the left language network (the AG1 subgroup). In this subgroup of the aphasic patients, there was a significant negative relationship between performance in reading comprehension and the RD of the right hemisphere TFexcF. It should be noted that all written stimuli used in this study involved regular words, and therefore our findings are discussed in relation to processes associated with the lexical route. This is not the first time that the so-called “minor” hemisphere for language has been shown to be associated with reading abilities. Gazzaniga (1970) in his publication “The Bisected Brain” reported that some split-brain patients were able to read and understand written nouns with their right hemisphere, which also seemed to have some limited grammatical capacity. In the left hemisphere, a region that includes the posterior occipitotemporal sulcus and extends medially and laterally to the adjacent fusiform and inferior temporal gyri has been found to activate during word reading (Price, 2000). It has been suggested that this region acts as “an interface between visual form information and higher-order stimulus properties, such as its associated sound and meaning” (Devlin et al., 2006). The posterior inferior temporal gyrus has also been found to contribute to early word recognition through interaction with the visual word form area in the fusiform gyrus (Dien et al., 2013). The inferior temporal gyrus in the left hemisphere is a critical part of the ventral language system processing high-level visual information and the middle temporal gyrus is critical for the processing of semantic information and both these temporal cortical regions communicate with Broca’s area in the inferior frontal gyrus, the key language production region, *via* the TFexcF (Petrides and Pandya, 2009; Petrides, 2014). Thus, high-level visual form processing that is integrated with semantic (meaning) information in the anterior to intermediate temporal region communicates with Broca’s area *via* the TFexcF.

The present results have also shown a significant negative relationship between speech fluency and the RD values of the TFexcF in the right hemisphere in the aphasic group with large LH damage (AG1 group). Speech fluency was measured with the use of two BDAE tasks: a. the Stroke Story (SS), which requires the patient to produce spontaneous speech based on biographical memory, and b. the Cookie Theft Picture (CTP), which requires the description of a visually presented scene during speech production. Previous research had significantly and negatively linked left TFexcF damage with performance on the Stroke Story, demonstrating the role of left hemisphere TFexcF in spontaneous, memory-related speech production (Efthymiopoulou et al., 2017).

It has been suggested that the left ventrolateral frontal region, and in particular Brodmann area 45 in the pars triangularis, plays a major role in the controlled selective retrieval of information from posterior cortical regions (Petrides, 2002, 2015) and there is good evidence for this role in functional neuroimaging studies (Cadoret et al., 2001; Kostopoulos and Petrides, 2016), including selective lexical retrieval (Grindrod et al., 2008). The comparable frontal regions in the right hemisphere are also activated during word retrieval in healthy participants (Vigneau et al., 2011), and a possible compensatory role of these regions after word retrieval deficits following a LH cerebrovascular accident has been proposed (Winhuisen et al., 2005). The findings of the present study are consistent with the role of the TFexcF in such processes.

The present study also demonstrated significant negative correlations of RD in the dorsal and ventral tracts (i.e., AF, SLF III, and TFexcF) with various aspects of language performance in the AG2 group. In this group, the lesions were markedly smaller and located in various cortical and subcortical regions (Figure 8, Table 2) and the patients exhibited moderate or mild aphasia (see Table 4). Specifically, the RD of the TFexcF in the RH was significantly correlated with performance in the PPVT-R, CIG, and BDAE simple command comprehension tests (see “Materials and Methods” section). In the PPVT-R word comprehension test, the examinee is asked to select the most appropriate visual stimulus (which could be an object, a living being, or an action) that matches best to a spoken word of increasing difficulty and abstraction (Simos et al., 2010). Thus, selective retrieval is required. Strategic selective retrieval from semantic memory in relation to verbal material has been linked to activation in the left inferior frontal gyrus and anterior temporal cortex (Moss et al., 2005; Grindrod et al., 2008), i.e., the regions connected *via* the TFexcF in the left hemisphere. Selective retrieval of visuospatial and nonverbal information is subserved by its right counterpart (Kostopoulos and Petrides, 2003), providing a possible explanation regarding the correlation between PPVT-R performance and RD of the TFexcF in the right hemisphere that was found in the present study.

The CIG and BDAE simple command comprehension tests require from the examinee to understand the meaning of an aurally presented sentence and either point to the correct stimulus (CIG test) or act according to the command (BDAE; Tsapkini et al., 2009; Simos et al., 2014). The participation of the RH in language comprehension has been supported both at the word level in split-brain and fMRI studies (Gazzaniga, 1970, 1998; Price, 2000) and at the sentence level (Kuperberg et al., 2000; Vigneau et al., 2011; Gajardo-Vidal et al., 2018). Kuperberg et al. (2000) provided fMRI evidence that the right middle temporal gyrus contributes to the processing of prosodic elements during sentence comprehension.

In the present study, performance in CIG (i.e., an auditory comprehension task with a working memory component; Simos et al., 2014) did not correlate only with the TFexcF but also with RH SLF III. Gajardo-Vidal et al. (2018) found that activation of the right IFG was related to both linguistic and non-linguistic working memory and lesion in this area in right-handed patients would result in long-lasting auditory sentence comprehension impairment. The present findings are in accordance with the notion that the right hemisphere IFG, which is connected dorsally *via* the SLF III and AF with posterior parietal and temporal regions and ventrally *via* the TFexcF with intermediate temporal regions, may mediate attentional/executive processes.

Note that repetition of words and sentences was also negatively correlated with the RD of both the AF and SLF III, as well as the TFexcF. Repetition ability involves a number of processes, such as phonemic analysis, articulation, short-term memory/attention, and lexical semantic knowledge (Ardila and Rosselli, 1992). In a study using transcranial magnetic stimulation, Hartwigsen et al. (2010) provided evidence that the right supramarginal gyrus (i.e., the posterior termination of SLF III) contributes to accurate phonological decisions in the healthy brain. Moreover, Hartwigsen et al. (2020) found that after applying ‘virtual lesions’ over left posterior IFG in post-stroke patients with left temporoparietal lesions, phonological decisions were delayed and this delay was correlated with the upregulation of the lesion homolog in the right hemisphere supramarginal gyrus, suggesting a possible role of right-lateralized homolog regions. Thus, the right hemisphere SLF III and AF linking supramarginal gyrus and posterior superior temporal regions, respectively, with IFG appear to participate in tasks involving phonemic processing, especially when the left SLF III is affected. The posterior part of left inferior frontal gyrus (i.e., pars opercularis) is typically linked to phonemic fluency (Schmidt et al., 2019), along with the left pars triangularis and left temporal lobe. Note that phonemic fluency in aphasia patients has been shown to depend not only on the integrity of the left inferior frontal cortex but also on its right homolog (Perani et al., 2003). The results of the present investigation are in line with the above findings, suggesting a role of the right posterior IFG and its connections in phonemic fluency.

Finally, a marginally significant correlation was observed between SLF III RD in the RH and performance in the Boston Naming Test (BNT). BNT besides articulation and visual perception involves lexical-semantic retrieval. Baldo et al. (2013) studied patients with left hemisphere lesions using voxel-based lesion analysis and, after controlling for deficits in visual recognition and motor speech, identified the mid-posterior middle temporal gyrus in the left hemisphere as the critical region for lexical-semantic retrieval. Similarly, in healthy adults, left posterior temporal areas have been identified as critical for naming (Hamberger et al., 2014). However, a broad network including left as well as right hemispheric areas, such as the right precentral gyrus and inferior temporal gyrus is found to be activated during the process of picture naming (Hamberger et al., 2014).

As discussed earlier, many fMRI studies have reported bilateral activations, often in right hemisphere homologs of language areas, during the performance of different language tasks in healthy participants (Price, 2000; Price et al., 2005; Price, 2012; Vigneau et al., 2011; Hamberger et al., 2014) and in aphasia patients (Perani et al., 2003; Hartwigsen et al., 2010, 2020; Baldo et al., 2013; Gajardo-Vidal et al., 2018). The present results demonstrating correlations between language performance and RH white matter tracts in both the AG1 and AG2 groups support the hypothesis that, when the left language networks are disrupted, RH homolog networks may be recruited for the compensation of specific language functions. Note that a different pattern of correlations was observed between the two aphasia subgroups, i.e., AG1 and AG2. This finding may be related to the difference in performance between the two groups (see “Results” section), the severity of aphasia (see Table 4), and lesion volume (see Table 2). In the subgroup in which the LH lesion was restricted to only part of the left language network (AG2; see Table 2), there were significant correlations of performance with both dorsal and ventral language tracts, but in the subgroup with large lesions (AG1), the correlations were only with RD in the ventral TFexcF.

In summary, the present study demonstrated structural differences between aphasic patients and healthy participants, in the form of increased AD, in the right hemisphere homologs of language-related tracts. Although it is possible that these differences reflect premorbid structural peculiarities in particular individuals, they may also reflect mechanisms triggered by the left-lateralized cerebrovascular accident. In any case, the underlying etiology of such differences was beyond the scope of the present study. The main aim of the present research was to examine possible relationships between structural white matter indices and language performance in patients with chronic aphasia due to a left hemisphere stroke. In this context, evidence is provided of significant inverse associations between the RD values in specific right hemisphere tracts of the aphasic patients and performance on specific language tests. The fact that the observed correlations included RD values that were not found to differ from those of the healthy participants supports the idea that premorbid variability in the myelination structure of these RH tracts may provide an adaptive advantage in the compensation of language function after disturbance of the left language network in certain individuals.

Thus, the present study provided evidence in patients with aphasia of an association between specific linguistic and anatomical variables related to homologs in the right hemisphere of white matter tracts that constitute the ventral and the dorsal streams for language in the language dominant left hemisphere. Notably. the present study is the first to relate the RH TFexcF with speech fluency and reading, indicating a possible compensatory role for this particular white matter structure in certain language functions, which appear to be related to the extent of damage in the LH.

Moreover, our findings suggest different association patterns between right TFexcF indices and language variables between the two aphasia subgroups. AG1 included the patients with more extensive lesions, who exhibited inferior scores on speech narration, as well as repetition and reading aloud tests, compared to AG2 that included the patients with more restricted damage. It is safe to assume that severe nonfluent speech output would compromise performance on the tests in which the participant had to respond verbally, by either reading aloud or repeating words/sentences. In this sense, the fundamental difficulty of the AG1 subgroup of patients when compared with the AG2 subgroup could be considered to be the ability to produce speech, which involves several processes, such as retrieval of semantic information, word selection, and phonemic construction. TFexcF indices were found to be related to speech narration tasks only for the AG1 subgroup. By contrast, in the AG2 subgroup, TFexcF, as well as SLF III and AF, indices were shown to be correlated with several language tasks, mostly related to comprehension of commands and repetition. In the case of AG2 (i.e., patients with more restricted lesions and significantly better abilities to produce speech), such scores could be considered to be affected by an executive capacity which involves immediate memory. We, therefore, speculate that in AG2, the right hemisphere tracts could support part of a domain-general, bilateral monitoring network, as mentioned in the discussion. On the other hand, in AG1, the TFexcF could be hypothesized to be the neurological substrate of a compensatory mechanism, which may be activated by the massive left perisylvian lesion, and attempts to support the processes underlying narration, which have been severely impaired. Taken together, the above arguments may point to a differential compensatory role of the right TFexcF in aphasia, which could be dependent on lesion extent and severity of aphasia.

## Data Availability Statement

The data analyzed in this study is subject to the following licenses/restrictions: the datasets generated during and/or analyzed during the current study are available from the corresponding author on reasonable request. Data cannot be available for open access since the informed consent signed by all participants does not allow their use from any organization other than the Eginition hospital. Requests to access these datasets should be directed to EKo, ekourtid@gmail.com.

## Ethics Statement

The studies involving human participants were reviewed and approved by Eginition Hospital Ethics Committee. The patients/participants provided their written informed consent to participate in this study.

## Author Contributions

EKo conceived and designed the study, performed neuropsychological testing, analyzed the data, performed DTI data analysis and white matter tract reconstruction, contributed to the interpretation of the results, and wrote the manuscript. DK performed neuropsychological testing, analyzed the data, participated in the processing of the T1 structural data and lesion reconstruction, contributed to the conception/design of the study, interpretation of the results, and writing of the manuscript. IZ and IE contributed to study conception and design and revised the manuscript. EKa performed the acquisition and analysis of the MRI data and revised the manuscript. GA contributed to acquisition and processing of speech data as well as to the statistical analysis of the DTI and speech data, conducted the processing of T1 structural data and lesion reconstruction, contributed to white matter tract reconstruction as a second rater, and revised the manuscript. GV performed the acquisition and analysis of the MRI data, and revised the manuscript. NK supervised the acquisition and analysis of the MRI data and revised the manuscript. CP contributed to study conception/design and interpretation of the results, and revised the manuscript. MP, who was the main supervisor of this study, contributed to study conception/design, interpretation of the results, and writing of the manuscript. MP supervised the placement of ROIs and verified the reconstructions of the white matter tracts. All authors contributed to the article and approved the submitted version.

## Conflict of Interest

The authors declare that the research was conducted in the absence of any commercial or financial relationships that could be construed as a potential conflict of interest. The handling Editor declared a past co-authorship with several of the authors GA, DK, GV, EKa, NK, CP.

## Footnotes

^1^http://www.mccauslandcenter.sc.edu/mricrogl/

## Abbreviations

AD: axial diffusivity
AF: arcuate fasciculus
AG1: aphasia group 1
AG2: aphasia group 2
BDAE-SF: Boston Diagnostic Aphasia Examination—Short Form
BNT: Boston Naming Test
CIG: Comprehension of Instructions test in Greek
CTP: Cookie Theft Picture
CVA: cerebrovascular accident
DTI: diffusion tensor imaging
FA: fractional anisotropy
fMRI: functional magnetic resonance imaging
MRI: magnetic resonance imaging
PPVT-R: Peabody Picture Vocabulary Test-Revised
RD: radial diffusivity
RH: right hemisphere
ROI: region of interest
SLF II: superior longitudinal fasciculus II
SLF III: superior longitudinal fasciculus III
SR: speech rate
SS: stroke story test
TFexcF: temporo-frontal extreme capsule fasciculus
UF: uncinate fasciculus.
