# Supplementary material for: The Role of the Right Hemisphere White Matter Tracts in Chronic Aphasic Patients After Damage of the Language Tracts in the Left Hemisphere
Source: Front Hum Neurosci. 2021 Jun 22;15:635750. doi: 10.3389/fnhum.2021.635750 (PMC8258417; doi:10.3389/fnhum.2021.635750)
Supplement: Supplementary file 1 [file Table_1.docx]

| *Table S1. Spearman rho correlation coefficients indicating the relationship between right hemisphere white matter FA, AD and RD values and neuropsychological performance* | | | | | | | | | | | | |
| --- | --- | --- | --- | --- | --- | --- | --- | --- | --- | --- | --- | --- |
| ***AG1*** | | | | | | | | | | | | |
|  | **TFexcF**  **FA** | **TFexcFAD** | **TFexcFRD** | **AF FA** | **AF AD** | **AF RD** | **SLF III FA** | **SLF III AD** | **SLF III RD** | **SLF II**  **FA** | **SLF II AD** | **SLF II RD** |
| **AC-W** | 0.503 | -0.221 | -0.651 | 0.221 | -0.393 | -0.405 | 0.368 | -0.160 | -0.233 | 0.651 | 0.061 | -0.307 |
| **AC-SC** | 0.422 | -0.169 | -0.542 | 0.096 | -0.530 | -0.337 | 0.253 | -0.241 | -0.193 | 0.651 | 0.253 | -0.241 |
| **AC-CM** | 0.491 | -0.172 | -0.651 | 0.160 | -0.307 | -0.307 | 0.393 | -0.110 | -0.258 | 0.565 | 0.110 | -0.258 |
| **Rp-W** | 0.577 | -0.196 | -0.675 | 0.307 | -0.417 | -0.503 | 0.454 | -0.246 | -0.356 | 0.602 | -0.110 | -0.295 |
| **Rp-S** | 0.764 | 0.062 | -0.764 | -0.452 | -0.436 | 0.171 | 0.078 | 0.031 | 0.171 | 0.047 | -0.109 | 0.031 |
| **Rd-W** | 0.779 | -0.153 | ***-0.899**** | -0.102 | -0.587 | -0.217 | 0.409 | -0.230 | -0.217 | 0.447 | -0.102 | -0.230 |
| **Rd-S** | 0.756 | 0.126 | -0.756 | -0.504 | -0.378 | 0.252 | 0.000 | 0.126 | 0.252 | 0.000 | 0.000 | 0.126 |
| **Rd-SCa** | 0.574 | -0.365 | -0.691 | 0.300 | -0.73 | -0.652 | 0.717 | -0.652 | -0.652 | 0.782 | -0.300 | -0.652 |
| **Rd-SCb** | 0.741 | -0.445 | ***-0.877**** | -0.037 | -0.692 | -0.321 | 0.507 | -0.321 | -0.321 | 0.494 | -0.161 | -0.222 |
| **COWF-ph** | 0.327 | 0.109 | -0.409 | -0.027 | -0.245 | -0.082 | 0.109 | -0.027 | -0.082 | 0.382 | 0.409 | -0.027 |
| **COWF-s** | 0.736 | 0.000 | -0.799 | -0.190 | -0.583 | -0.127 | 0.292 | -0.203 | -0.127 | 0.431 | 0.038 | -0.203 |
| **BNT** | 0.395 | -0.168 | -0.551 | 0.156 | -0.335 | -0.299 | 0.228 | -0.060 | -0.132 | 0.611 | 0.311 | -0.156 |
| **PPVT-R** | 0.143 | -0.143 | -0.257 | -0.086 | -0.257 | 0.029 | -0.371 | 0.486 | 0.543 | 0.543 | 0.600 | 0.314 |
| **CIG** | 0.340 | -0.454 | -0.605 | 0.094 | -0.359 | -0.189 | 0.265 | -0.019 | -0.094 | 0.624 | 0.246 | -0.113 |
| **SS** | 0.761 | -0.101 | ***-0.824***** | -0.114 | -0.685 | -0.254 | 0.419 | -0.355 | -0.254 | 0.507 | -0.140 | -0.355 |
| **CTP** | 0.761 | -0.101 | ***-0.824***** | -0.114 | -0.685 | -0.254 | 0.419 | -0.355 | -0.254 | 0.507 | -0.140 | -0.355 |
| ***AG2*** | | | | | | | | | | | | |
|  | **TFexcF**  **FA** | **TFexcFAD** | **TFexcFRD** | **AF FA** | **AF AD** | **AF RD** | **SLF III FA** | **SLF III AD** | **SLF III RD** | **SLF II**  **FA** | **SLF II AD** | **SLF II RD** |
| **AC-W** | 0.211 | -0.199 | -0.130 | 0.434 | -0.004 | -0.392 | 0.435 | -0.032 | -0.309 | 0.405 | -0.013 | -0.334 |
| **AC-SC** | 0.071 | -0.437 | ***-0.641**** | 0.368 | -0.464 | -0.560 | 0.450 | -0.377 | -0.548 | 0.309 | -0.252 | -0.492 |
| **AC-CM** | -0.037 | -0.290 | -0.446 | 0.254 | -0.240 | -0.576 | 0.248 | -0.323 | -0.565 | 0.334 | -0.271 | -0.546 |
| **Rp-W** | 0.165 | -0.481 | ***-0.699**** | .576 | -0.146 | ***-0.664^*^*** | 0.602 | -0.294 | ***-0.632^*^*** | 0.521 | -0.237 | -0.511 |
| **Rp-S** | -0.063 | -0.172 | -0.374 | 0.202 | 0.035 | ***-0.704**** | ***0.741**** | -0.001 | ***-0.768**** | 0.067 | -0.101 | -0.185 |
| **Rd-SCa** | 0.009 | -0.175 | -0.154 | 0.069 | -0.142 | -0.253 | 0.145 | -0.096 | -0.202 | 0.018 | -0.151 | -0.193 |
| **Rd-SCb** | 0.145 | -0.313 | -0.222 | 0.308 | -0.120 | -0.215 | 0.251 | -0.072 | -0.190 | 0.195 | -0.104 | -0.226 |
| **COWF-ph** | -0.069 | -0.263 | -0.514 | 0.188 | -0.301 | -0.480 | 0.382 | -0.289 | ***-0.593^**^*** | 0.126 | -0.375 | -0.418 |
| **COWF-s** | 0.061 | -0.329 | -0.390 | 0.174 | -0.318 | -0.308 | 0.386 | -0.107 | -0.401 | -0.059 | -0.306 | -0.249 |
| **BNT** | -0.020 | -0.361 | -0.523 | 0.294 | -0.349 | -0.474 | 0.434 | -0.286 | ***-0.555^**^*** | 0.025 | -0.424 | -0.397 |
| **PPVT-R** | -0.254 | -0.209 | ***-0.693**** | 0.335 | -0.623 | -0.614 | 0.321 | -0.636 | ***-0.662^*^*** | 0.315 | -0.571 | -0.693 |
| **CIG** | 0.073 | -0.426 | ***-0.748**** | 0.302 | -0.628 | -0.606 | 0.204 | -0.508 | ***-0.604^**^*** | 0.075 | -0.539 | -0.524 |
| **SS** | -0.012 | -0.081 | -0.010 | -0.051 | 0.098 | -0.054 | -0.007 | 0.100 | -0.032 | -0.201 | -0.049 | 0.091 |
| **CTP** | 0.007 | -0.130 | -0.029 | 0.042 | 0.074 | -0.125 | -0.017 | 0.120 | -0.017 | -0.184 | -0.056 | 0.059 |
| **p_adjusted_ <0.05, **p_adjusted_ <0.055 (marginally significant)*  *Abbreviations: AF: Arcuate Fasciculus; AD: axial diffusivity; FA: fractional anisotropy; RD: radial diffusivity; SLF II, Superior Longitudinal Fasciculus II; SLF III, Superior Longitudinal Fasciculus III; TFexcF, Temporo-Frontal extreme capsule Fasciculus*  *AC-W: BDAE Auditory comprehension – single words; AC-SC: BDAE auditory comprehension –simple commands; AC-CM: BDAE Auditory comprehension – complex material; Rp-W: BDAE word repetition; Rp-S: BDAE sentence repetition; Rd-W: BDAE single word reading; Rd-S: BDAE Sentence reading; Rd-SC: BDAE sentence comprehension; COWF-ph: Controlled Oral Word Fluency phonemic subscale; COWF-s: Controlled Oral Word Fluency semantic subscale; BNT: Boston Naming Test; PPVT-R: Peabody Picture Vocabulary Test-Revised; CIG: Comprehension of Instructions in Greek.* | | | | | | | | | | | | |
